# Salience-Based Selection: Attentional Capture by Distractors Less Salient Than the Target

**DOI:** 10.1371/journal.pone.0052595

**Published:** 2013-01-28

**Authors:** Michael Zehetleitner, Anja Isabel Koch, Harriet Goschy, Hermann Joseph Müller

**Affiliations:** 1 Department Psychologie, Ludwig-Maximilians-Universität München, Munich, Germany; 2 Graduate School of Systemic Neurosciences, Ludwig-Maximilians-Universität München, Planegg-Martinsried, Germany; 3 Department of Psychological Sciences, Birkbeck College, University of London, London, United Kingdom; University of California, Davis, United States of America

## Abstract

Current accounts of attentional capture predict the most salient stimulus to be invariably selected first. However, existing salience and visual search models assume noise in the map computation or selection process. Consequently, they predict the first selection to be stochastically dependent on salience, implying that attention could even be captured first by the second most salient (instead of the most salient) stimulus in the field. Yet, capture by less salient distractors has not been reported and salience-based selection accounts claim that the distractor has to be more salient in order to capture attention. We tested this prediction using an empirical and modeling approach of the visual search distractor paradigm. For the empirical part, we manipulated salience of target and distractor parametrically and measured reaction time interference when a distractor was present compared to absent. Reaction time interference was strongly correlated with distractor salience relative to the target. Moreover, even distractors less salient than the target captured attention, as measured by reaction time interference and oculomotor capture. In the modeling part, we simulated first selection in the distractor paradigm using behavioral measures of salience and considering the time course of selection including noise. We were able to replicate the result pattern we obtained in the empirical part. We conclude that each salience value follows a specific selection time distribution and attentional capture occurs when the selection time distributions of target and distractor overlap. Hence, selection is stochastic in nature and attentional capture occurs with a certain probability depending on relative salience.

## Introduction

Visual attention can be allocated in a stimulus-driven (bottom-up) or an observer-guided (top-down) fashion [1], with both sources of control combining to determine which location or object in the field is attended. The process of selection often is investigated in the realm of visual search. In this paradigm, the task is to find a pre-defined target among distractors and (depending on the task) indicate its presence or absence or make another decision based upon its features. Attentional selection in the search process has been subject to a variety of experimental studies [2]–[5] as well as computational models [6]–[10].

A variant of the visual search paradigm that permits attentional selection to be investigated precisely is the visual search distractor paradigm [11], [12]. In this paradigm, a task-relevant target singleton and an irrelevant distractor singleton (both carrying unique features compared to all other stimuli) are surrounded by homogeneous non-target stimuli. An example would be a display containing a predefined target, a grey tilted bar, and a distractor, a colored vertical bar, amongst grey vertical non-target bars. The task is to find the target while ignoring the distractor. Typically, the item with the highest feature contrast is selected first or ‘captures attention’ initially, as evidenced by reaction time (RT) interference (for distractor-present compared to -absent trials) when the distractor is characterized by a higher feature contrast (relative to the non-targets) than the target [3], [11]–[16], but not when it has a lower feature contrast [11]–[13]. On this basis, it has been claimed “that the initial shift of attention [is directed] to the most salient singleton” [3] and “that the bottom-up salience signal of the stimuli in the visual field determines the selection order” [3].

In terms of functional architecture, stimulus-driven selection in visual search is thought to be mediated by an attention-guiding ‘master’ [17], ‘activation’ [6], or ‘salience map’ [18]–[20], which codes the physical distinctiveness of each location in the field in terms of its total feature contrast against the surrounding locations: the more a stimulus differs from those in its surround (e.g. a bar tilted by 45°, as compared to 7°, amongst vertical bars), the stronger its salience signal. A winner-take-all mechanism then selects that location on the salience map for focal-attentional allocation which exhibits the highest level of activation. In terms of the computations involved, existing models assume that after low-level feature extraction, a center-surround algorithm returns contrast images for each feature channel; these feature contrast maps are later combined to form the feature-independent salience map, which serve as the basis for the attentional selection mechanism [18]. Although, in principal, attention is guided to the location with the highest activation, salience models typically assume noise to influence some stage(s) of salience computation [19], [20]. Noisy coding turns selection into a stochastic process: the more salient the target, the higher the probability that it is the first item selected. The assumption of noise influencing attentional guidance is shared by prominent models of visual search [6], [8], [10], [21].

Noise turns computed salience into a random variable with a certain distribution and an expected value. Consequently, these models require a differentiation of the concept of ‘*salience*’: salience may refer (i) to the expected value of the distribution of salience estimates, which corresponds to the distinctiveness of each item from its surround, as captured by contrast images or image statistics [22]–[24]; or (ii) to the actual outcome of the salience computation process on a given trial, which is subject to variability (due to noise) and can thus deviate from the expected value. To illustrate this differentiation, it is instructive to linken salience-based selection to motion (direction) discrimination treated as a decision process [25]. Discrimination of motion direction within random dot kinematograms is a frequently used paradigm in the modeling of decisions [26]. Typically in this paradigm, some 100 dots are moving within a bounded area (some 3° of visual angle in diameter): a proportion of dots move coherently to either the left or the right, while the remaining dots have random trajectories. The observer's task is to indicate the direction of the coherent motion. The decision model [25] presupposes the existence of motion-sensitive cells whose rate of firing is proportional to the coherence of motion in a specific direction. For the left versus right decisions, the relevant cells are those tuned to leftward and, respectively, rightward motion within their receptive fields. Hence, when a patch of dots is presented with a proportion of dots moving coherently e.g. to the right, signal detection models of this decision assume that the cells of both types exhibit activity, which is noisily distributed around different means. In particular, with rightward coherent motion in a random dot kinematogram, the activity induced in ‘right cells’ would be distributed around a mean value greater than that of the activity induced in ‘left cells’. The higher the proportion of coherently moving dots in the display, the farther apart the means of the two activity distributions are. A decision could be made by drawing one sample of evidence from the ‘left’ unit and one from the ‘right’ unit, choosing that direction which shows a higher level of evidence [27]. Decision models that do not only describe the outcome of decisions (as is the case with signal detection models), but also the distribution of decision times assume that the noisy activity of the motion-sensitive cells is integrated, or accumulated, over time. The output of this accumulation process, the decision variable, is constantly compared against a decision criterion, until the decision is made. That is, the noisy activity of motion detectors (e.g. in MT) is accumulated into a decision variable (presumably in the lateral intraparietal sulcus, LIP), based on which the decision is made.

We propose a similar logic for salience-based selection. Instead of two motion detectors for the two relevant directions in a random-dot motion discrimination task, we posit salience detectors for each location of visual space which are sensitive to feature contrast. These detectors have previously been assumed to be noisy. Instead of a signal detection theory-based decision, such as in Guided Search 2.0 [10], we propose that each detector's activity is accumulated into a decision variable over time. All these decision variables are constantly compared against a criterion, with the first accumulator whose activity reaches the criterion leading to attentional selection of the respective location. Accordingly, this model of selection does not only describe the outcome, but also the time course of selection decisions. That is, salience-based selection, rather than being taken to consist of the two successive steps, namely ‘salience computation’ followed by ‘attentional selection’, is considered as dynamic process in which a noisy signal is accumulated over time that triggers a selection decision.

Thus, as becomes apparent from the above considerations, there are two conceptually different notions of salience. The construct of physical feature contrast, which corresponds to motion coherence in the random dot kinematogram, is represented as sensory data by the activity of salience detectors in the brain (analogous to the activity of motion detectors representing motion coherence). This momentary neural representation is distributed around its mean, that is, it is a noisy signal. Because the expected salience value, that is the mean of the neural salience representation, is not linearly related to physical feature contrast [28], [29], it needs to be estimated. This estimation is the intent of current salience models [22]–[24]. However, relevant for selection on a given trial is the accumulated signal of the neural representation, which is the decision variable. For clarity, in the remainder of the article, we refer to the concept of expected salience value as *stimulus salience* and the actual or accumulated estimate as *selection salience*, because the latter is the basis for attentional selection on a given trial. Stimulus salience is related to physical stimulus properties: for instance, a horizontal bar among vertical bars has a higher stimulus salience than a bar tilted by 30°. Solely based on the value of stimulus salience, focal-attentional selection would have to favor the horizontal bar. However, owing to noise in the computation process, the resulting estimates (i.e. selection salience) are distributed around the expected value of stimulus salience. Hence, if the distributions of selection salience for horizontal and 30° orientation contrasts overlap, first selection of the 30° bar is possible in principle: the selection salience of the 30° bar can be higher on a given trial than that of the horizontal bar. Stimulus and selection salience do not usually have to be differentiated in standard visual search (detection) tasks with only one salient target being present – because, despite noise, the stimulus salience distributions of target and non-targets virtually never overlap and the selection salience of a non-target can never be higher than that of the target. However, this differentiation becomes important when two conspicuous stimuli are presented, but only one is task-relevant: if selection salience is higher for the irrelevant (distractor) stimulus, even though its stimulus salience is lower than that of the relevant (target) item, it will nevertheless be attentionally selected first.

Thus, because of the noisy salience computation, in the distractor visual search paradigm, attentional capture would occur when the distractor has a higher selection salience than the target. A distractor can have a higher selection salience if its stimulus salience is higher, equal, or even lower compared to that of the target, depending on the overlap between the distributions of the target's and the distractor's selection salience. Consequently, (i) the occurrence of attentional capture would be proportional to the relative stimulus salience of the target and the distractor and (ii) distractors even less stimulus salient than the target would capture attention in a proportion of trials. This implies that if the proportion of attentional capture events is high, RT interference would be large; and if it is low, interference would be small.

Note, however, that this hypothesis has never been tested directly. Most studies of attentional capture have used only singleton distractors that were more salient than the target [14], [30]–[33], and so cannot address this issue at all. On the other hand, there are a few studies that have contrasted (at most) two stimulus salience conditions [11]–[13], [34]. But even then, one cannot logically make any inferences about the stochastic dependency of selection (order) on stimulus salience (quite apart from the fact that interference effects heavily depend on the sample that is drawn from all possible stimulus salience values, that is the studies with two settings are likely to have contrasted only extreme, low and high, values of stimulus salience). In other words, although salience and visual search models assume noise in the selection process accounting for attentional capture by less stimulus-salient distractors, there is, to our knowledge, as yet no empirical evidence for this assumption. Testing this assumption would require varying the salience of targets and distractor parametrically, rather than (just) dichotomically.

On this background, the present study was designed to test the hypothesis of stochastic dependency between stimulus salience and attentional selection [10], [21], using a combined approach of behavioral evidence and quantitative modelling [18]–[20]. In the behavioral part, we parametrically manipulated the stimulus salience of pop-out targets and pop-out distractors – so as to be able to (i) examine the occurrence of attentional capture across a greater range of stimulus salience values and (ii) determine the quantitative relationship between stimulus salience and attentional selection, that is, selection salience. For achieving these aims, it was necessary to quantify the difference in stimulus salience between targets and distractors – which we did by means of a visual search go/no-go *detection* task in which each of the pop-out stimuli, whether it served as a target or a distractor in the visual search *distractor* task, was presented as a single, to-be detected pop-out stimulus (i.e., without an irrelevant pop-out stimulus being present in the display). The detection RTs measured in this task served as estimates for stimulus salience. The difference in stimulus salience between a given target-distractor pair in the visual search *distractor* task was then quantified in terms of the difference in their associated detection RTs when they were presented alone in the visual search *detection* task. This procedure permitted us to compare stimulus salience across different dimensions.

Given that noise in the salience computation process turns attentional selection into a stochastic process, we expected (i) RT interference to be dependent on the relative stimulus salience and (ii) even less stimulus-salient distractors (compared to the target) to interfere, that is capture attention, in some proportion of trials. By contrast, if salience is not a random variable, as suggested by some authors [11], [12], or noise is too small to affect attentional selection between two salient stimuli, attentional capture should occur only with distractors more stimulus-salient than the target. In order to verify that RT interference by less salient distractors is indeed caused by attentional capture, we recorded eye movements in an additional experiment with distractors less salient than the target.

As a second step, we computationally modeled the results of the behavioral visual search distractor experiment; specifically, we modeled selection salience in the distractor paradigm based on the stimulus salience parameters estimated from the behavioral data in the detection task (see also [35]). The model we implemented is based on two-stage models of visual search, which assume that stimulus salience is computed spatially in parallel for all items in the display (stage 1) and then focal attention is allocated to the item with the highest selection salience value (stage 2). Note, that our model only describes the first step of this process: the salience-based decision as to what location in space attention should select. The second step, including attentional engagement and stimulus identification, is outside the scope of the present model. The only model that (to our knowledge) has made the distinction between stimulus salience and selection salience explicit is Guided Search [10]. GS assumes that the selection salience value is stochastically related to stimulus salience, that is pre-attentive salience coding for each item in the display is subject to noise, necessitating a signal-detection-type decision [36] as to which item to transfer to the second, focal-attentional processing stage. Signal detection models, in general, account for response proportions, such as those of hits and false alarms, but not for the temporal duration of the underlying decisions. Likewise, GS makes statements only about the proportion of selection decisions directed to the target versus to a non-target, but not the time-course with which the decisions are made. However, pop-out targets can differ in the speed with which they are singled out, that is they can be equivalent in terms of selection proportion (the target is always selected first), but differ in the time it takes until the item is selected. Behaviorally, it has been demonstrated that targets that pop out (i.e., that have flat RT/set-size functions) can differ in detection RTs [37]–[40]. For example, among vertical bars, both a target tilted by 45° and one tilted by 12° pop out, but differ in their associated detection RTs. Töllner, Zehetleitner, Gramann, and Müller [41] demonstrated that such differences in RTs are indeed attributable to differences in selection times: the latency of the so-called N2pc component of the EEG, which is assumed to reflect the transition from pre-attentive to post-selective stimulus processing [42], [43], increased as a function of decreasing stimulus salience of the pop-out target. Given this finding and the notion that a selection decision is based on the accumulated sensory evidence [25], we considered it important to take into account the time course of selection decisions in our model; that is, we simulated the data of the visual search distractor paradigm in a new model of salience-based selection that assumes a time course of selection decisions and thus permits the proportion of capture trials to be predicted for a given salience difference (derived from the respective detection RTs) between target and distractor.

In summary, the present study had two goals, one empirical and one theoretical. Empirically, it was designed to test two central predictions of visual search and salience models: in a distractor paradigm, (i) RT interference should be proportional to the difference in stimulus salience between target and distractor, and (ii) interference should also be observed with distractors less stimulus-salient than the target. Furthermore, assuming that this RT interference is actually caused by attentional capture (rather than some filtering cost [44]) less stimulus-salient distractors should also be found to capture the eyes. Theoretically, the study was intended to computationally model the conceptual distinction between stimulus salience (as estimated by RTs in a search detection task without distractors) and selection salience, the noisy estimate of stimulus salience computed by the pre-attentive visual system. To this end, the data of the behavioral visual search distractor experiment were modeled, based on the behaviorally estimated stimulus salience parameters. The model makes predictions about which item is selected first, rather than about RT interference.

## Behavioral Reaction Time Experiment

### Methods

#### Ethics statement

Participants gave their written informed consent. The study was approved by the ethics committee of the Department of Psychology, LMU Munich, in accordance with the Code of Ethics of the World Medical Association (Declaration of Helsinki).

#### Participants

Fifteen paid (€ 16) volunteers, with a median age of 27 (range 20–50) years, five of them male, all dextral and with visual corrected-to-normal acuity, participated in this study.

#### Stimulus presentation and data acquisition

The experiment was conducted in a sound-insulated room, and was controlled by a program purpose-written in C++. Stimuli were presented on a 19″ View Sonic Graphics Series G 90 fB monitor at a resolution of 1,024×768 pixels and a refresh rate of 85 Hz; viewing distance was approximately 57 cm. Participants responded using their left and right index fingers, respectively, to press one of two vertically arranged buttons on a purpose-built response pad. RTs and response accuracy were recorded online.

The display consisted of 39 vertical broken grey bars presented on black background and arranged on three imaginary concentric circles (1.88°, 3.25°, and 4.63° of visual angle in radius, with 8, 12, and 18 bars, respectively) around the center of the screen, which was occupied by another bar. Bars were 0.25°×1.13° in size and had a 0.13°-gap randomly located at the top or the bottom of each bar. Targets differed from non-targets in orientation (7, 8, 9, 14 and 45° tilted from vertical), and distractors differed from non-targets in luminance (13.8, 14.8, 17.9, 19.4, and 25.5 cd/m^2^ for distractors and 5.25 cd/m^2^ for non-targets). A pilot experiment was conducted to ensure that target and distractor salience was sufficient for these stimuli to ‘pop out’ from the search array, that is, their associated detection times were independent of the number of non-targets in the display (see Text S1 and Table S1).

#### Design and procedure

Two 1-hr sessions were carried out on consecutive days, at the same time of day. The first part of each session was the distractor experiment; the second part was a post-experiment for stimulus salience measurement (for the latter, see *Baseline salience measurement*). The within-subject design of the distractor experiment was 2 (distractor present vs. absent)×5 (target salience)×5 (distractor salience) factorial, resulting in 25 salience difference conditions. A target was present on all trials; a distractor occurred randomly in 50% of the trials. Target and distractor were placed randomly at the 12 possible positions on the second circle to keep eccentricity constant. All salience difference conditions were presented in random order within blocks. Participants completed 20 blocks of 50 trials each day, yielding a total of 2,000 trials and 40 trials per salience difference condition.

**Figure 1:**
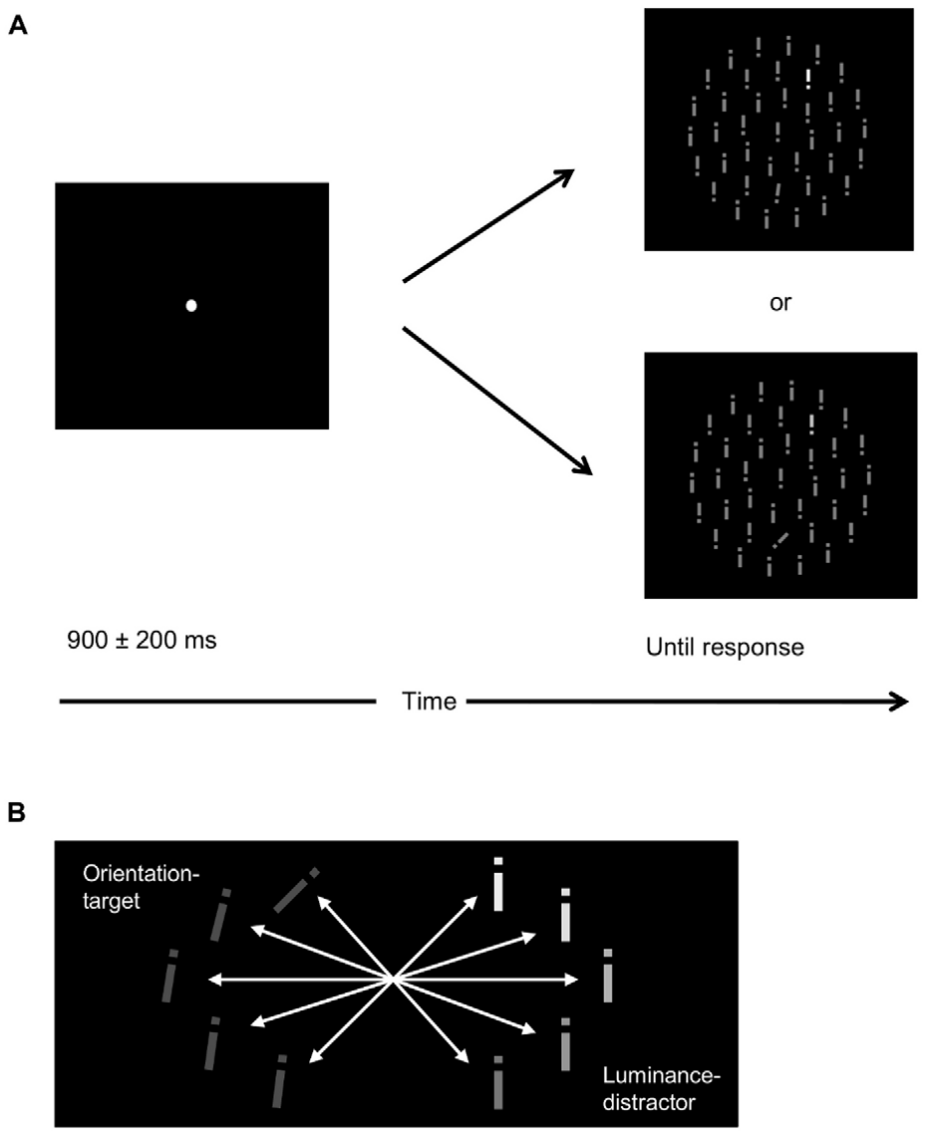
Experimental design and stimuli. (a) A search display, consisting of 39 broken grey bars arranged around three imaginary concentric circles, was presented in the center of the screen, on a black background. There was always an orientation target; and in half of the trials (randomly determined), there was also a luminance distractor. Each trial started with a white fixation spot that was hidden while the display was presented until response. Inter-stimulus-intervals varied randomly in the range 900±200 ms. While ignoring a bright distractor, participants searched for a tilted target bar and decided, via a speeded button press, whether the gap was located at the top or the bottom of the bar. This response decision required focal attention to be allocated to the target. (b) 25 Salience difference conditions resulted from 5 orientation (7, 8, 9, 14, 45°) and 5 luminance (13.8, 14.8, 17.9, 19.4, and 25.5 cd/m^2^) contrasts.

Each trial started with a white fixation dot (radius = 0.05°) presented for a duration uniformly distributed between 900 and 200 ms, that was superseded by the search display which remained present until response (Figure 1A). Participants were instructed to indicate, as quickly and accurately as possible, the gap location (top or bottom) of the target by pressing the upper or lower button, respectively. In case of an error, visual feedback was provided, followed by an additional 500-ms blank screen before the next trial. At the end of each block, participants were informed about their mean RT and error rate.

#### Baseline salience measurement

Because salience is not linearly related to physical contrast [29], we used a behavioral measurement of salience, which was collected in a post-experiment after each session of the distractor experiment. Stimuli were the same as in this experiment. All target orientation and distractor luminance contrasts from the distractor experiment (Figure 1B) were presented as (to-be-detected) targets randomly intermixed with target-absent displays (as in the distractor experiment, targets never occurred on the outer circle). The design was 2 (target presence vs. absence)×2 (dimension luminance vs. orientation)×5 (contrast) factorial. Dimensions were blocked, contrasts were mixed within blocks. Participants' task was to indicate the presence of an orientation or luminance target via button press; response was to be withheld if no target was present. Four blocks consisting of 80 trials were performed each day, yielding a total of 640 trials and 32 trials per contrast condition. The stimulus display was presented until response or a maximum of 1,200 ms. Error feedback was provided visually, immediately after the false response.

Using these detection RTs as our measure of stimulus salience, we calculated the salience difference between stimuli by subtracting distractor salience from target salience. For example, if a target was detected at a rate of 300 ms and an distractor at a rate of 400 ms, then their salience difference was −100 ms. Note that items of higher salience are associated with shorter RTs; negative salience differences indicate a distractor less salient than the target, and positive differences a distractor more salient than the target. This salience difference measure served as independent variable in the distractor experiment.

#### Data analysis

Only correct-response trials were used for analysis (distractor experiment: 96.5%; baseline salience measurement: 99.0%), excluding RTs shorter than 150 and longer than 1,500 ms in the distractor experiment (0.8%) and shorter than 150 and longer than 1,000 ms in the baseline salience measurement (0.2%). The first 20 trials (first 10 trials of the baseline salience measurement) of each session and the first 3 trials of each block served as practice trials and were also excluded from analysis. RT interference was calculated by subtracting mean RTs for target-only trials from mean RTs for target-plus-distractor trials. Statistical data analysis was carried out with R software [45]. Regression analyses were conducted with *n* = 25 salience difference conditions (aggregated across 15 participants); t-tests for RT interference of less salient distractors were conducted with *n* = 15 participants.

To test for the dependency of RT interference on relative salience between target and distractor, we used nonlinear least-square estimation for regression function fitting. The nonlinear function followed the form:
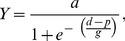
(1)where *a* is the asymptote or maximum RT interference, *d* the salience difference, *p* the inflection point, and *g* the growth factor of the function.

Goodness of fit comparison of the regression functions was carried out using Bayes Information Criterion [46], which is calculated according to
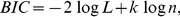
(2)where *L* is the maximum likelihood of the data under the regression function, *k* the number of parameters to be estimated, and *n* the number of observations. Smaller *BIC* values indicate a better model fit.

### Results and Discussion

**Figure 2:**
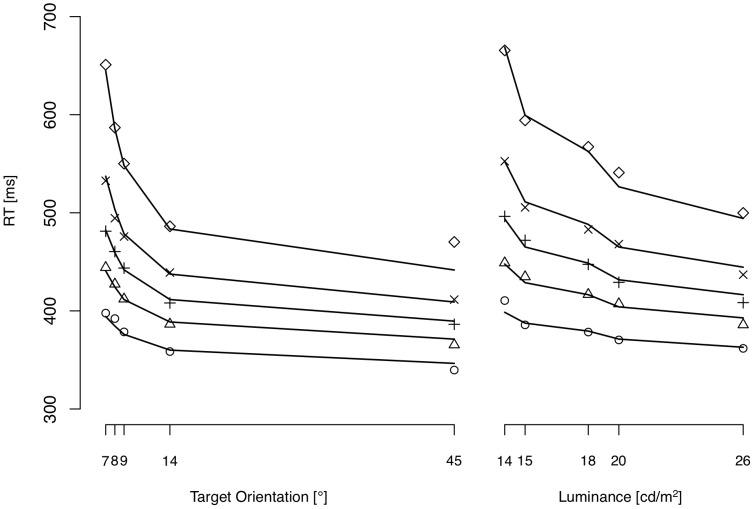
Empirical data of the baseline salience measurement and data fitted by the accumulator salience model. Left panel: five salience levels of orientation targets. Right panel: five salience levels of luminance targets. Symbols depict RT quantiles of each condition as follows: o = .1, Δ = .3, + = .5, × = .7, and ◊ = .9. Lines represent RTs generated by the model. Fitted RTs differ from empirical RTs by 5 ms on average (range: 0 to 28 ms). Additional parameter estimates were *T*_er_ = 300 ms, *s*_er_ = 70 ms, a = .08, and β = .294.

We investigated the order of attentional selection in a distractor experiment with a unique, orientation-defined pop-out target present on all trials and a unique, luminance-defined pop-out distractor randomly interspersed in half the trials (Figure 1A; for stimulus pop-out characteristics, see Text S1 and Table S1). Target orientation and distractor luminance were manipulated such that the salience difference between the two items was varied parametrically in 25 steps (Figure 1B). Stimulus salience was estimated in a post-experiment (*Baseline salience measurement*) in which no distractors were presented and targets could be defined in the orientation or the luminance dimension. The times required to detect these targets served as salience estimates for the stimuli in the distractor experiment (Figure 2). We used the mean salience difference values of all participants to predict RT interference on distractor-present, compared to distractor-absent, trials using nonlinear regression functions. RT interference in this task is commonly attributed to automatic prior selection of the distractor, and absence of interference to direct selection of the target [12].

**Figure 3:**
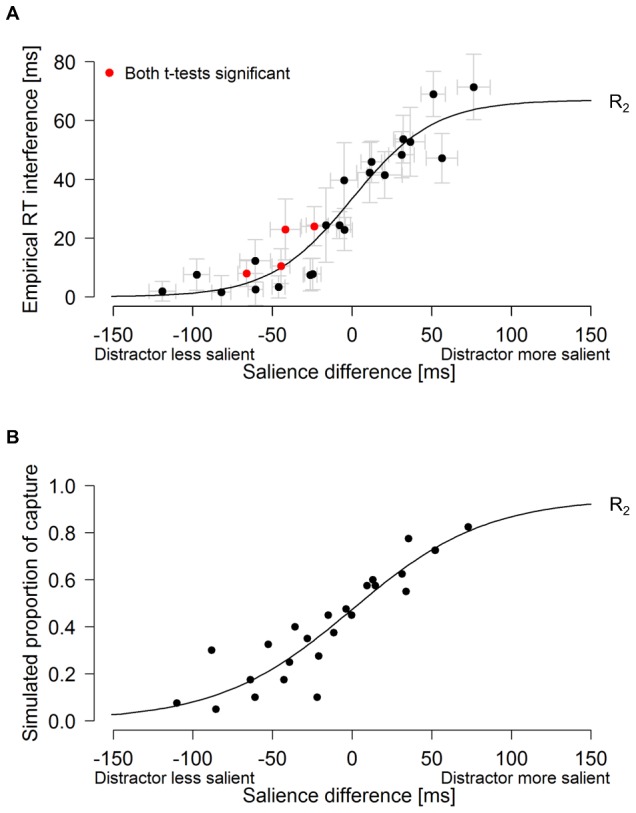
Behavioral interference and modeled proportion of capture as a function of salience difference. (a) Empirical RT interference, averaged across participants, represents the RT difference, in ms, between distractor-present and distractor-absent trials. Salience difference, averaged across participants, was derived from detection times in the baseline salience measurement requiring a simple target-present vs. target-absent decision (see *Methods* of *Behavioral reaction time experiment*). Negative x-values indicate distractors less salient, and positive x-values distractors more salient than the target. Dots represent mean values of RT interference for each salience difference condition (*n* = 25); arrows indicate the associated standard errors. Red dots indicate significant RT interference by distractors significantly less salient than the target (t-tests: *p*<.05). Solid curve: regression function curve *R_2_*. (b) Proportion of capture in the distraction experiment was predicted by salience difference, derived from fitting empirical salience difference values. Again, dots represent mean values of RT interference for each salience difference condition (*n* = 25). The curve depicts the nonlinear relationship according to *R*_2_.

Figure 3A presents the observed RT interference (for correct-response trials), averaged across participants (mean RT [± *SEM*] on distractor-present trials = 660 [±12.9] ms; mean RT interference = 28 [±4.4] ms), for luminance-defined distractors and orientation-defined targets as a function of their salience difference. RT interference was strongly correlated with the salience difference (*n* = 25; Pearson's *r* = .91 [*t*(23) = 10.8, *p*<.001]), indicative of the order of selection (‘target first’) being dependent on relative object salience. This relationship already exhibits the expected characteristics: (i) the magnitude of interference varies with the salience difference between target and distractor, and (ii) distractors considerably less salient than the target do interfere with search.

Next, we fitted two nonlinear regression functions to the data, one with the inflection point free to vary (*R_1_*) and one in which it was fixed to 0 ms salience difference (*R_2_*). We then compared the functions' goodness of fit by examining their Bayes Information Criterion values [46], where smaller *BIC* values indicate a better fit. Regression function *R_1_* yielded an asymptote of 73 ms, an inflection point of 7 ms, and a growth factor of 29 ms. For the nonlinear regression function *R_2_*, where the inflection point was set to 0 ms, the RT interference asymptote was estimated to be 67 ms, and the growth factor to be 26 ms salience difference. *BIC* value comparison confirmed regression function *R_2_* (with the inflection point set to 0 ms) to fit the data better than *R_1_* (*BIC*_R1_ = 178 vs. *BIC*_R2_ = 175; see Table 1 for details).

**Table 1:** Parameter estimates of the model predictions fitted to empirical and modeled data.

Variable	Unstandardized estimate	*S.E.*	*t*	*p*	*CI*	*BIC*
*Human data*						
*R*_1_						178
Asymptote	73	9.66	7.53	**.001**	58–117	
Inflection point	7	10.26	0.65	.263	−10–48	
Growth factor	29	5.79	5.79	**.019**	18–47	
*R*_2_						175
Asymptote	67	2.90	23.09	**<.001**	61–73	
Inflection point	0					
Growth factor	26	3.39	7.57	**<.001**	19–34	
*Model data*						
*R*_2_						−42
Asymptote	0.95	0.04	24.87	**<.001**	0.87–1.03	
Inflection point	0					
Growth factor	42	5.01	8.39	**<.001**	32–55	

*Note: n* = 25. Estimate for empirical data in ms; asymptote estimate for modelled data in proportions. *R*_i_ = Nonlinear regression function. *S.E.* = Standard Error. *t* and *p* = value and probability of the t statistic associated with parameter estimate. Degrees of freedom: R_1_: 23, R_2_: 22. *CI* = 95% confidence interval. *BIC* = Bayes Information Criterion.

**Figure 4:**
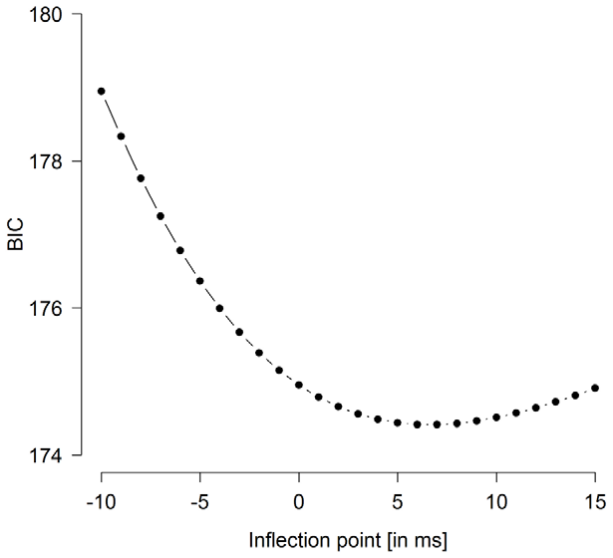
Course of BIC dependent on the inflection point of the regression function. Regression functions were fitted according to formula (1), with the inflection point as fixed parameter. Inflection points are specified in ms of salience difference.

These results argue in favor of a proportional first selection of the distractor dependent on its salience difference to the target. The function where the inflection point was set to 0 ms indicates that equally salient targets and distractors are equally likely (50%) to be selected first. First-selection probability for a given item then increases as its relative salience increases. The shift of the inflection point into the positive range in regression function *R_1_* indicates that at the point at which selection probability is equal for both items, the target is actually less salient than the distractor (rather than the two stimuli being equi-salient). This might reflect an influence of top-down control, permitting the target to compensate for this discrepancy in relative salience. However, reconsidering our measure of relative salience, it is possible that target and distractor salience is not the same in the distractor experiment as measured in the baseline salience measurement. There are three possibilities of how they may differ between tasks. First, if a stimulus is presented alone as in the baseline salience measurement, the display is more homogeneous compared to when an additional distractor is presented – in which case salience might be overestimated in the baseline salience measurement relative to the distractor experiment. However, because this would apply to both the target and the distractor, this should not affect relative salience in the distractor experiment. A second reason for diverging relative salience in the distractor experiment derives from the fact that stimulus salience was measured after the distractor experiment. One might argue that assigning the role of target to the orientation dimension (and that of distractor to the luminance dimension) in the distractor experiment induces ‘priming’ for orientation-defined singletons, resulting in an overestimation of target salience and an underestimation of distractor salience in the subsequent baseline salience measurement. The implication is that at 0 ms salience difference, the distractor would actually be more salient than the target and the true point of equal salience would lie in the negative range of salience differences. However, according to Maljkovic and Nakayama [47], priming effects for the orientation dimension, as an aftereffect of having been assigned the target role in the distractor experiment, should dissipate within a few trials in the baseline salience measurement. Third, stimulus salience might be different in the distractor experiment because of top-down weighting [48]–[51]. When both stimuli are presented together, as in the distractor experiment, the weight of the target might be up-modulated and that of the distractor down-modulated. That is, the salience values determined in the baseline salience measurement would be under-estimates for targets and over-estimates for distractors. If this was the case, true equality of salience should be in the positive range of salience differences and the distractor would be even less salient than the target at the point of 0 ms salience difference. To test for the latter two possible types of salience estimation errors, we fitted regression functions with varying inflection points from −10 to 15 ms salience difference and calculated the corresponding *BIC's*. As figure 4 shows, *BIC* was lowest for a regression function with the inflection point in the positive range of salience differences. This implies that at 0 ms salience difference, in the distractor experiment, the distractor is still less *stimulus-salient* than the target and top-down weighting shifts the point of equal salience difference into the positive range. Consequently, our measure of salience difference is rather conservative, that is RT interference by less salient distractors is actually even higher than we have assumed here.

The nonlinear regression function already implies that distractors less salient than the target do interfere with search. To examine RT interference by less salient distractors more closely, we conducted t-tests for all salience differences for which the distractor was significantly less salient (criterion of 0 ms salience difference) than the target. These tests confirmed there are indeed distractors less salient than the target that produced significant RT interference (Figure 3A).

Overall, the findings of RT interference being sigmoidally related to relative salience and of less salient distractors capturing attention, are compatible with visual search and salience models [10], [18]–[24] that assume that the salience coding and, thus, the selection process is subject to internal noise.

### Computational Model

A second, theoretical goal of the present study was to develop and test a computational model of how stimulus salience translates into selection salience, that is, a model accounting for the variation in the outcome of the selection process based on stimulus salience – concretely by simulating the data of the distractor paradigm. Importantly, the model we devised makes predictions about the item that is selected first (rather than directly about RT interference) and takes noise and the time course of selection, based on stimulus salience, into account. Selection is assumed to involve a decision between all stimuli in the display and the dynamics of selection processes to be stochastic in nature [10], [19]–[24], with the outcome being dependent on stimulus salience and a noise component.

**Figure 5:**
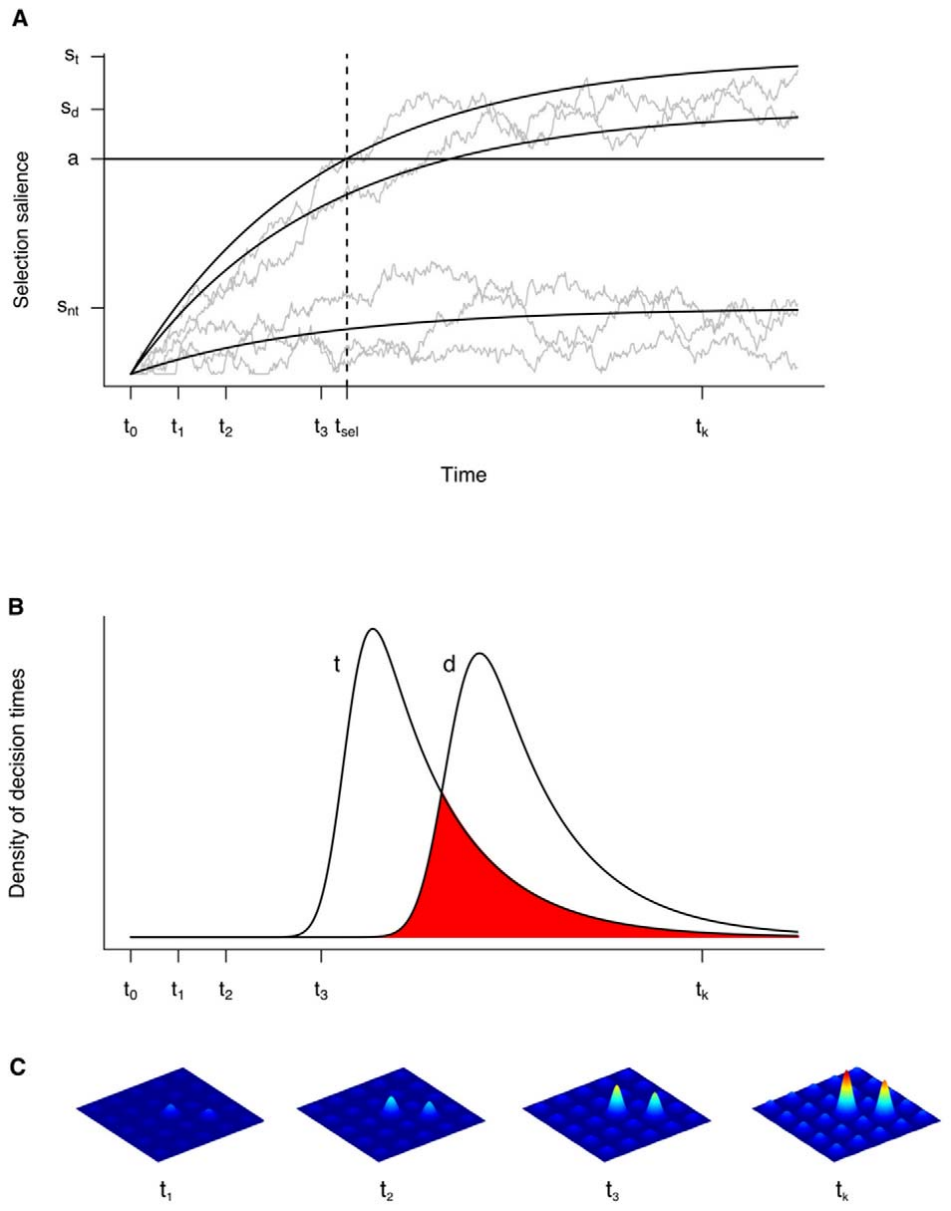
Stochastic model of salience-based selection. (a) For each location in the visual field, salience is accumulated over time t = {t_1_, t_2_,…, t_k_} by leaky accumulators. Gray jagged lines represent sample paths of sensory evidence accumulation over time, influenced by noise. Mean accumulation behavior is indicated by solid black lines. Salience asymptotes *s* (s_t_ = target salience, s_d_ = distractor salience, s_nt_ = non-target salience) indicate maximum salience when time is infinite and noise absent; asymptotes correspond to the salience values of map locations computed by deterministic models. (b) Selection time distributions (t = target, d = distractor) indicate selection time variation due to noise. Overlap of these distributions (red area) marks the range within which a distractor may be selected first even if it is less salient than the target. (c) The final salience pattern evolves over time, as illustrated by heat maps at different points in time.

In more detail, the model assumes that the salience map develops over time probabilistically (Figure 5). Each item in the visual scene is represented by a sensory-evidence accumulator unit, the drift rate of which corresponds to stimulus salience. Accumulation is assumed to be a leaky and noisy process [52]. That is, sensory evidence does not accumulate infinitely, but comes to settle eventually around an asymptotic value (mathematically the proportion of the drift rate to leak). A selection decision is triggered as soon as sensory evidence for a specific location exceeds a threshold. In this model, stimulus salience determines the drift rate with which sensory evidence is accumulated, and selection salience is the accumulating, or accumulated, sensory evidence. In contrast to this dynamic process, which is continuous over time, conventional models of visual salience essentially envisage a snapshot-like topographic representation of the (physical) feature contrasts present in the scene, which serves as the basis for selection decisions: the location of maximum contrast is attentionally selected by a winner-take-all mechanism, the time course of which is usually not modeled explicitly.

For simulating the results of the distractor experiment, in a first step, we fitted the model to the empirical baseline salience measurements in order to obtain parameter estimates for stimulus salience; in the next step, these parameters were used to simulate selection salience in terms of the probability of a distractor versus a target being selected first.

### Methods

We implemented the selection salience map using leaky accumulators [52]. That is, all items on the screen are represented by leaky accumulators that race against each other for selection; the item that first exceeds a threshold criterion is then selected. Model parameters are drift rate ν, leakage *β*, and threshold *a*. At each time step, sensory evidence of accumulator *I* is updated according to the formula:
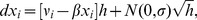
(4)where h is the step size, which is set to 1 ms in the model fits, and *Ν*(0, σ) denotes a Gaussian distribution with mean 0 and standard deviation σ. Within-trial variability is normally distributed with σ = 0.1. Salience computation terminates as soon as one accumulator exceeds the selection threshold, resulting in a decision time of attentional selection (t_sel_). Observed reaction time is usually considered to be the sum of decision time and time of non-decision-related processes such as basic encoding time between retina and primary visual cortex as well as the time necessary after the decision has been made for the motor commands to be transmitted to and innervate the effector muscles. Non-decision times (denoted *T*_er_), which incorporate the time necessary for stimulus encoding and response production, are usually assumed to be distributed uniformly [53] with range *s*_er_. Note that, potentially, the model could also be turned into a winner-take-all ‘network’ by adding lateral inhibition between each accumulator. In this case, over time, there would eventually be only one accumulator active, with the activities of all other accumulators driven to (near-) zero. As concerns the selection times for the first item, the main question at issue in the present study, such a model would yield similar results.

In pop-out search, accumulators for non-target stimuli can be left out of the simulation, because non-targets are effectively never selected – as evidenced by search time for pop-out targets being independent of the number of non-targets [38]. That is, for the baseline salience measurement, in which only a target (but no distractor) was presented amongst the non-targets, the selection salience map model is reduced to one accumulator racing towards its threshold. In the distractor experiment, by contrast, a pop-out target and a pop-out distractor were presented simultaneously. In the model, this is represented by two accumulators racing against each other, with the drift rates of the two accumulators corresponding to target and distractor stimulus salience, respectively.

The simulation proceeded in two steps: first, the model described above was fit to the data of the baseline salience measurement to obtain drift rates corresponding to the different levels of stimulus salience induced by the 10 possible ‘targets’, as well as estimates of the other parameters (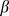, *a*, *T*_er_, and *s*_er_); second, these estimated parameters were then used to simulate the proportion of capture trials in the distractor experiment.

From the empirical data of the baseline salience measurement, RT distributions were characterized by the .1, .3, .5, .7, and .9 quantiles. These were calculated per observer per condition and then pooled across all observers [53]. Model parameters consisted of one selection threshold *a*, leakage *β*, non-decision time *T*_er_ and its range *s*_er_, and additionally one drift rate ν_i_ per salience condition. For each parameter set, 50,000 replications of the random walk process were simulated (see equation 4); that is, for each salience condition, the model produced 50,000 model RTs. From these, the model .1, .3, .5, .7, and .9 quantiles as well as the error rates were computed. An error was recorded if the accumulator failed to reach the selection threshold within 1,200 ms (as in the empirical experiment). For each parameter set, the weighted least squares (WLS) was calculated according to
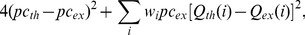
(5)where *pc* stands for percent correct and the indices *th* and *ex* denote the modeled (*th*eoretical) and empirically measured (*ex*perimental) statistics, respectively; *Q(i)* signifies the .1, .3, .5, .7, and .9 quantile RTs, and *w_i_* is a weight which was set to 2 for the .1 and .3 quantiles, to 1 for the .5 and .7 quantiles, and to 0.5 for the .9 quantile [54]. That is, the squared differences between empirical and model percent-correct scores and, respectively, empirical and model quantiles are calculated, and the latter differences are weighted more strongly for lower than for higher quantiles, because estimates for higher (especially the .9) quantiles are more variable than those for ‘faster’ quantiles. A Nelder-Mead simplex optimization algorithm [55] implemented in R [45] was used to minimize the WLS cost function. The fitting procedure commenced with manually selected starting variables and was run for 200 iterations ten times in a row, each time using the optimization result from the previous run as starting values for the next run in order to avoid local minima. Local minima are likely to be avoided by this procedure, because during the simplex optimization, the step sizes with which the parameter space is sampled become adaptively smaller. When restarting the algorithm, the step size is increased again, thus providing the potential for escaping from a local minimum [56]. Finally, the optimization procedure was run with maximally 5,000 iterations to yield the final set of parameters.

Those parameters which fitted best to the baseline salience measurement data were then used to simulate capture in the distractor experiment. The model was based on the assumption that in cases of both, a target and a distractor being present, two accumulators race against each other for selection, one with a drift rate corresponding to target stimulus salience and the other with a rate corresponding to distractor stimulus salience; the accumulator which first reaches the selection threshold wins the race. Capture was then operationalized as the proportion of trials in which the distractor accumulator completed the race before the target accumulator. For each combination of target and distractor, the selection threshold *a*, the leakage 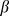, and the two salience values were taken from the fit of the baseline salience measurement data and 40 races were simulated (the same number of trials as were used in the empirical study).

### Results and Discussion

As RT interference is an indirect measure of the order of attentional selection, the underlying mechanism can only be inferred. Therefore, to strengthen our hypothesis about the relationship between salience and order of selection, we computationally implemented the proposed salience-based selection mechanism (Figure 5), estimated target salience from the (behavioral) baseline salience measurement, and simulated interference for the distractor experiment. RTs generated by the salience model yielded a close fit to the empirical RT distributions (Figure 2) for the various orientation and luminance targets in the baseline experiment: reduced salience slowed search and increased the spread of the RT distributions. The goodness of fit is remarkable given that across the ten different target conditions, only one parameter (the drift rate, corresponding to salience) was free to vary, whereas the parameters *a* (selection threshold), *β* (leakage of the accumulator), *T*_er_ (non-decision time), and *s*_er_ (variability of *T*_er_) were kept constant.

Importantly, when simulating the data of the distractor experiment using the fitted parameters from the baseline salience measurement, the predicted proportions of capture were similar to the observed RT interference (Figure 3B): the salience model simulates distractors less salient than the target to capture attention, the proportion of capture events to depend sigmoidally on salience difference, and capture to occur in half the trials with distractor of equal salience relative to the target. This qualitative similarity is reinforced by comparing the fits of nonlinear regression function *R_2_* to the simulated and the empirical data: the inflection point and growth factor parameters of the nonlinear fits did not differ, as indicated by the overlapping confidence intervals (see Table 1). Keeping the leakage parameter *β* constant at zero does not qualitatively alter the fit of the baseline experiment or the proportions of interference. However, there are two conceptual arguments for assuming leakage. First, without leakage, evidence would accumulate towards infinity over time, which is implausible with respect to the limitedness of neuronal firing rates. Second, with leakage, sensory evidence averages to an asymptote which is proportional to the salience values calculated by conventional, ‘static’ salience algorithms.

## Behavioral Eye Movement Experiment

Although RT interference has been attributed to attentional capture in most previous studies [11]–[13], [15], there is also the possibility that RTs are slower on distractor compared to target-only trials not because attention is first captured by the distractor, but because the distractor draws on the same processing resources as the target and thus slows target selection. Conceivable mechanisms of slowing are filtering [57] or competitive interactions [58] to be resolved in favor of the target. Whatever the precise mechanism that may underlie such slowing effects, in the present context, the critical question is whether or not the RT interference produced by distractors less salient than the target is the result of attentional capture. Empirical RT data cannot answer this question (RT interference may be caused by slowing, attentional capture, or both), and although our modeling results demonstrate that a capture account could explain the pattern of RT interference effects, it does not rule out alternative accounts in terms of non-capture slowing. Given this, we examined for attentional capture of the eye by (less salient) distractors in an eye-tracking experiment. Involuntary capture of the eye by a distractor is commonly taken as a strong indicator of attentional capture [59]. Accordingly, the finding of oculomotor capture would corroborate attentional capture as a source of RT interference. In the eye-tracking experiment, participants' task was to make a direct saccade to the target, while a less salient distractor could be present in the display.

### Methods

Methods were the same as in the RT distractor experiment, unless stated otherwise.

#### Participants

Eight paid (€ 8) volunteers, with a median age of 23 (range 20–39) years, one of them male, seven dextral, and with visual corrected-to-normal acuity and normal color vision, gave written informed consent to participate in this experiment.

#### Stimulus presentation and data acquisition

Stimuli were generated using a ViSaGe system (Cambridge Research Ltd., UK) with a purpose-programmed Experimental Toolbox for MATLAB (The MathWorks, Inc.). Stimulus displays were presented on a 22-inch Mitsubishi Diamond Pro 2070SB CRT monitor with a screen refresh rate of 120 Hz and a screen resolution of 1,024×768 pixels. Eye movements were recorded at a sampling rate of 1000 Hz by means of an EyeLink 1000 Desktop Mount eye tracker (SR Research Ltd., Canada) positioned below the display monitor. Participants viewed the monitor from a distance of about 70 cm; to minimize head movements, a chin and forehead rest were used. Eye movements were recorded from the right eye; however, stimulus displays were viewed binocularly.

Grey vertical bars (without gaps) of 0.25°×1.35° of visual angle were arranged on three imaginary concentric circles (2°, 4°, and 6° of visual angle in radius, with 6, 12, and 18 bars, respectively). Targets differed from non-targets in orientation (22° tilted from vertical, randomly to the right or left), and distractors differed from non-targets in color (distractor 1: 180/100/106, distractor 2: 171/104/110 RGB). All stimuli were matched for luminance.

#### Design and procedure

The experimental session started with the eye-tracking experiment, after which the baseline salience measurement was conducted. The eye-tracking experiment implemented a 2 (distractor absent vs. present)×2 (distractor salience) factorial within-subject design, with two salience difference conditions. To ensure reliable differentiation between target and distractor fixations for the data analysis, distractor positioning was restricted in the following way: the target position was chosen randomly out of the 12 possible positions on the middle circle; the distractor position was then chosen to be shifted by three or five positions to either the left or the right from the target position (each in a random 25% of the distractor-present trials). There were 80 trials per salience condition. This resulted in 320 trials overall, which were presented in 4 blocks of 80 trials each. All salience difference conditions were presented in random order within blocks.

The task was to make a speeded saccade to the target. Observers were instructed to fixate the fixation cross at the trial start until the appearance of the search display, and then to make a direct saccade to the (orientation) target, while ignoring the (color) distractor. In case the first saccade went nevertheless to the distractor, participants were instructed to direct the next eye movement to the target. In addition, they were told that after having made a saccade to the target, they should fixate it until the disappearance of the search display.

Each trial started with a fixation cross (0.5°×0.5°) for 1,000 ms. Then, the search display appeared and remained visible for 1,000 ms. The intertrial interval, in which a black (blank) screen was displayed, was of a random duration between 700 ms and 1,100 ms. Observers were encouraged to use this interval for briefly closing and resting their eyes, so that they could minimize blinks during the subsequent trial. Additionally, participants could take short breaks between experimental blocks. Prior to each block of trials, a nine-point calibration of the eye tracker was conducted.

#### Baseline salience measurement

Salience measurement was the same as for the reaction time experiment, unless stated otherwise. Apparatus and stimuli were the same as in the eye-tracking experiment, that is, the to-be-detected targets were either ‘oriented’ or ‘colored’. Six blocks consisting of 40 trials were performed, yielding a total of 240 trials and 40 trials per target condition. Each trial started with the presentation of a white fixation cross (0.5°×0.5°) for a random duration ranging from 700 ms to 1,100 ms. Thereupon, the search display was presented and remained visible until response or a maximum duration of 1,000 ms.

#### Data analysis

For the analysis of the baseline salience measurement, error trials (0.9%) and target-absent trials were excluded. In addition, RTs shorter than 150 ms and longer than three standard deviations above an observer's mean per target type were discarded as outliers (0.8% of all trials). For the analysis of the eye-tracking data, trials were excluded on which search display onset occurred during a saccade or the eye-tracker failed to track the observer's pupil (4.3%). Saccade latencies were calculated as the time between onset of the search display and the initiation of the observer's first saccadic eye movement. Trials with initial saccade latencies below 80 and above 600 ms were excluded (2.9%). The remaining data underwent a drift correction: Before the onset of the search display (i.e. at the end of the fixation cross display), gaze was assumed to have rested on the fixation cross. Thus, for drift correction, the eye's deviation from the fixation cross was subtracted from the subsequent gaze position data for this trial. The initial saccade after search display onset was then assigned to the target or the distractor if it landed within 3° of visual angle of the respective (target or distractor) location. Initial saccades that went neither to the target nor to the distractor were not included in the subsequent analysis (2.8% of the remaining trials).

Salience difference, which again served as independent variable, was computed as in the RT distractor experiment. To ascertain that each distractor was less salient than the target in the baseline experiment and whether the percentages of distractor fixations were greater than zero in the eye-tracking experiment, one-sided t-tests were calculated on the sample of eight participants.

### Results and Discussion

The eye-tracking experiment was designed to examine whether the interference by less salient distractors observed in the RT distractor experiment was the result of attentional capture; participants' task in this experiment was to make a speeded saccade to the orientation-defined target, while a color-defined, but less salient distractor could be present at the same time. Distractor color was manipulated in two steps. As in the RT experiment, stimulus salience was estimated in a post-experiment (*baseline salience measurement*). The times required to detect these (orientation- and color-defined) stimuli served as salience estimates for the stimuli in the eye-tracking experiment.

Detection times were significantly faster for the orientation target (*M* = 376 ms; *SD* = 37) compared to both color distractor 1 (*M* = 399 ms, *SD* = 54; *t* [7] = −2.1, *p*<.05) and color distractor 2 (*M* = 414, *SD* = 54; *t* [7] = −3.3, *p*<.01). Hence, both distractors were considerably less salient than the target.

**Figure 6:**
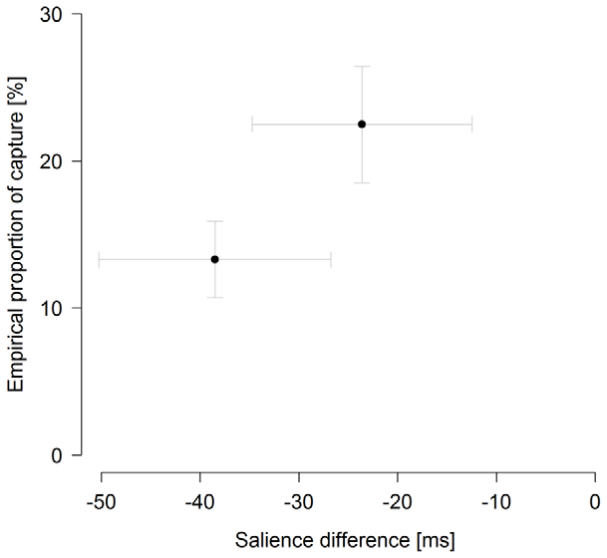
Capture of the eye by less salient distractors. Empirical proportion of capture by the distractor, averaged across participants, represents the proportion of first eye movements landing on the distractor position. Salience difference, averaged across participants, was derived from detection times in the baseline salience measurement requiring a simple target-present vs. target-absent decision (see *Methods* of *Behavioral eye-tracking experiment*). Negative x-values indicate distractors less salient than the target. Dots represent mean values of proportion of capture for each salience difference condition (*n* = 2); arrows indicate the associated standard errors.

For the eye-tracking experiment, we calculated mean percentages of target and distractor fixations (based on distractor-present trials) for the two distractor types. Figure 6 presents these as a function of the salience difference between target and distractor. With color distractor 1 (salience difference of −24 ms) in the display, 22.5% of the initial saccades went to this distractor rather than to the target. With color distractor 2 (salience difference of −39 ms), there were 13.3% oculomotor capture trials. The capture rate was significantly above zero for color distractor 2 as well as for color distractor 1 (*t* [7] = 5.1, *p*<.001 and, respectively, t[7] = 5.7, *p*<.001). Thus, even though both color distractors were less salient than the target (as established in the baseline salience measurement), they led to a considerable amount of capture events. This implies that distractors less salient than the target do give rise to involuntary attentional capture (as well as distractors more salient than the target).

The initial saccade latencies, irrespective of saccade destination, were examined in an ANOVA with the single factor distractor condition (three levels: absent, color distractor 1, color distractor 2). The latencies were somewhat shorter for distractor-absent trials (*M* = 249 ms, *SD* = 20) than for trials with a color distractor (distractor 1: *M* = 256 ms, *SD* = 29; distractor 2: *M* = 256 ms, *SD* = 31), but these differences were not reliable (*F* [2], [14] = 1.9, *p* = .19). The same was true when only the latencies of initial target fixations were examined: latencies were slightly, but not significantly, shorter for distractor-absent trials (*M* = 249 ms, *SD* = 20) than for trials with a color distractor (distractor 1: *M* = 259 ms, *SD* = 31; distractor 2: *M* = 256 ms, *SD* = 34; *F* [1], [9] = 2.4, *p* = .16, Greenhouse-Geisser-corrected).

Finally, we examined how long the eyes rested on the distractor when it was selected prior to the target. The mean fixation duration was 131 ms for color distractor 1 and 154 ms for color distractor 2. The 95% confidence intervals ranged from 95–160 ms for color distractor 1 and from 95–214 ms for color distractor 2. This means that the time required to identify the foveated item as a non-target and to prepare the next saccade varied between 95–214 ms.

This time can be related to the maximum RT interference in the behavioral distractor experiment. There, the asymptote of the sigmoidal relationship between salience difference and RT interference was about 80 ms. That is, distractors much more salient than the target, which are presumably selected first in 100% of all trials, lead to RT interference of approximately 80 ms. This time is in a similar range (albeit somewhat faster) to the durations of first fixations on distractors. Note, though, that the focus of the present study and model is on the capture of attention, rather than the subsequent processing steps which include identification of the selected item as a distractor, selection of the next salient location, disengagement of attention, and execution of the covert or overt attention shift. These processing stages subsequent to attentional capture have only rarely been discussed in the literature [3] and should be the subject of future research. The two methods presented here (fixation durations and maximum RT interference) may serve as two possibilities of how to estimate the duration of the subsequent processing stages.

## General Discussion

Theories of attentional selection, such as salience and visual search theories [10], [18]–[21], assume attention to be automatically attracted by the most salient location. An additional assumption of these theories is noise operating during the computation process. This assumption of noise requires the distinction between stimulus salience, determined by physical stimulus properties, and the salience estimate for selection which is susceptible to noise – selection salience. Although there are empirical studies providing evidence for attentional capture by the most salient stimulus [11], [12], [14], there has been no previous study in which salience of a target and salience of a distractor were varied parametrically, to demonstrate that noise influences the process of selection between two competing locations and turns salience into a stochastic variable such that even less salient stimuli lead to RT interference because they may be selected prior to the most salient ones. Note that the assumption of noise influencing the selection process is also at the heart of the redundant-signals paradigm. Here, two salient features share the same location while racing for selection [60], [61].

The aim of the present study was to test the predictions by visual search and salience models that noise influences the selection process such that (i) selection salience (based on which a selection is made) varies as a function of relative salience between target and distractor and (ii) distractors less stimulus-salient than the target capture attention. Further, by implementing the distinction between stimulus and selection salience computationally, we aimed at modelling the empirical results of the distractor visual search experiment.

By manipulating stimulus salience of targets and distractors parametrically, we found distractor interference to be sigmoidally related to salience difference between targets and distractors and even distractors less salient than the target to interfere with search and capture attention. These results are in accordance with salience [18]–[20] and visual search models [8], [10], [21], which assume noise during the selection process. This, at the same time, suggests that experimental manipulations of previous studies [11], [12], [14], [16] were insufficient to recognize the stochastic dependency between salience and attentional capture and hence claimed that the most (stimulus-) salient item is invariably selected first. Parametric salience manipulation, by contrast, revealed a gradual increase of RT interference with increasing distractor salience relative to the target, where a less salient distractor can be selected before the more salient target. These results point to a stochastic relationship between stimulus salience and selection, which is predicted by visual search and salience models, but was not shown in relevant empirical studies [11], [12], [14], [16].

### Attentional Selection as Decision Process

For the computational implementation of the distinction between stimulus and selection salience, we considered attentional selection as a decision between the target and the distractor (non-targets were considered negligible in the competition for selection, because it was ensured that all target and all distractor stimuli were found efficiently, i.e. popped-out) and used decision mechanisms to model selection salience on the basis of stimulus salience. The idea to implement attentional selection as a decision process is grounded on the assumption that search does not involve a one-step decision [62]–[64], but rather a chain of decisions [10], [65], [66]. In this chain, first, one of *n* possible locations has to be selected (where *n* is the number of possible target locations in the display); second, a two-alternative identification decision between ‘target’ and ‘distractor’ has to be made; third, a decision concerning the response-relevant feature (here the gap location) is necessary for task completion; and fourth, the correct button has to be selected for the response (here upper or lower).

As input for the selection salience modelling, we used the stimulus salience estimates measured in the detection experiment. Selection salience was then computed by the race of the two accumulators of target and distractor with their drift rates corresponding to the stimulus salience of both stimuli. In other words, the model was first fit to the RT distributions in the salience baseline measurement, which was designed to provide estimates of the drift rate parameters corresponding to the stimulus salience values of the various (orientation and luminance) target stimuli. This procedure of taking empirical data as input for the model to simulate visual search performance was also used by Purcell et al. [35]. When, second, using these empirical stimulus salience parameters to simulate the data of the distractor experiment, the proportion of simulated capture (i.e. trials on which the distractor was selected first) did not differ from that of empirical RT interference and increased with increasing relative stimulus salience between target and distractor. The model also simulated capture by less salient distractors, as indicated in the RT distractor experiment and demonstrated in the eye-tracking experiment.

The present approach of considering salience-based attentional selection as decision process (with a decision being made in favor of the stimulus with the highest selection salience), is only one way to conceive of salience. An alternative approach is that adopted by image-based salience models [22]–[24], which implement the construct of salience in terms of image statistics that are computed by center-surround algorithms. In this case, however, the most salient item is invariably selected, unless some noise filter is added on top of the computed salience. For the computation process itself, stimulus salience and selection salience are always identical in these models, that is, noise is not an inherent component of the computation process, but a ‘technical’ add-on following the computation of salience. A more theoretical, rather than technical, approach was taken in developing cognitive concepts of salience to explain specific patterns in visual search performance [10], [17], [21]. Here, the core function of salience (or activation) maps is their role in guiding attention to a specific location. Another perspective that has been taken to consider salience is the neurophysiological one [19], [67]–[70]. Here, the spike rates of neurons in the lateral intraparietal area or the frontal eye field are considered to form a salience map and marking locations for focal-attentional allocation. Some attempts have already been undertaken to combine the various constructs of salience: Li [20] presented a salience model based on neuronal network modelling of V1 that combines the cognitive, neurophysiological, and image statistics perspectives. Purcell et al. [35] combined the decision with the neurophysiological approach by feeding neuronal spike trains as salience signal to a stochastic accumulator model that simulated a decision in a visual search task. The variety of perspectives from which salience can be considered demonstrates that when various studies talk about salience, the authors do not necessarily have the same concept in mind. Thus, clearly, it is necessary to precisely define the concept under consideration, in order to frame predictions about behavior.

### Relation to Biased Competition

As mentioned above, RT interference due to distractor presence can have several possible sources. Either the distractor could capture attention and the target would be selected only as the second item, yielding a cost on RT; or the distractor could slow down selection of the target, even if the target is selected first. Here, we discuss these two theoretical possibilities with respect to the concept of biased competition [58], [71], [72]. The core assumption of biased competition is the idea that stimuli compete for neuronal representation. The competition for this representation can be biased by both top-down (intentional) and bottom-up (environmental) factors. The bottom-up factor relevant in this context is salience [73]: the more salient a stimulus is, the stronger it competes for neural representation. There are two possibilities of how this account can be linked to the distractor visual search paradigm.

First, biased competition could account for no-capture slowing of target selection. Target and the distractor compete for neural representation. Thus, when a distractor is present, fewer resources are available for the target. Even if the target is selected first, its selection time would be slower in the presence, compared to the absence, of a distractor. This could be implemented in our model in terms of lateral inhibition between the different accumulators [52], [74]. That is, each accumulator receives excitatory input from the salience signal derived from its stimulus and, additionally, inhibitory input from the other accumulators. However, while such a wiring scheme would implement the biased-competition mechanism sketched above, our eye movement experiment yielded little indication that the time required for direct (first) selection of the target is dependent on distractor presence (or distractor salience).

Second, our decision model – which assumes an accumulator for each stimulus in the visual field, with the drift rate of each being proportional to the stimulus salience – can be considered as an implementation of the bias in competition imposed by stimulus salience [73]. In the model, competition takes the form of a race, amongst the accumulators, against a threshold: that item is attentionally selected that drives the accumulator which crosses the threshold first, where the driving input depends on stimulus salience.

In summary, both variants of biased competition (yielding target slowing and distractor capture, respectively) can be implemented in our salience decision model. However, our eye movement data suggest that primarily the latter mechanism is responsible for the RT interference caused by a competing distractor, whether the distractor is more or less salient than the target.

### Relation to Top-down Modulations of Salience

The focus of the present study was on bottom-up modulations of salience by physical feature contrast. Top-down modulations of salience are well documented in the literature [10], [48], [50] and have also been discussed in relation to attentional capture [49], [75], [76]. Specifically, it is assumed that when a dimension (e.g. orientation) is task-relevant, salience signals from this dimension are up-modulated to some degree. At the same time, salience signals from irrelevant or to-be-ignored dimensions (e.g. luminance or color) are down-modulated. Our data support this view, in that the salience difference at which the target and the distractor are equally likely to be selected actually requires the distractor to be somewhat more salient than the target, as measured in the baseline experiment (because in the distraction experiment, top-down weights enhance the target and reduce the distractor salience). The present model can easily be extended to incorporate top-down weighting: the drift rates would be slightly increased for features in the target-defining dimension and decreased for features in the distractor-defining dimension, implementing task-dependent top-down modulations of salience.

### Conclusion

We conclude that attentional selection can be understood as a ‘decision’ and, consequently, with regard to the concept of salience, a distinction has to be made between *stimulus salience*, which is computed from physical stimulus properties, and *selection salience*, which contains the noisy estimate of stimulus salience that is relevant for attentional selection. Following this distinction, the dependency between attentional selection and salience is stochastic in nature. As an empirical consequence, attentional capture by an irrelevant distractor occurs as long as the selection time distributions of target and distractor overlap, and distractors less salient than the target can also capture attention.

## Supporting Information

Text S1 **Pilot Experiment.**
(DOCX)

Table S1 **Slopes and intercepts of the search RT functions for orientation and luminance contrast conditions.**
(DOCX)
