# Supplementary material for: Salience-Based Selection: Attentional Capture by Distractors Less Salient Than the Target
Source: PLoS One. 2013 Jan 28;8(1):e52595. doi: 10.1371/journal.pone.0052595 (PMC3557287; doi:10.1371/journal.pone.0052595)
Supplement: Text S1 — Pilot Experiment. (DOCX) [file pone.0052595.s001.docx]

# **Text S1**

# **Pilot Experiment**

A pilot experiment for stimulus calibration was conducted, to ensure that targets and distractors ‘pop out’ from the background irrespective of the number of surrounding stimuli. Only if search time is independent of set size, one can assume parallel processing and attentional allocation to be dependent on salience signals [1]. In this experiment, we tested a range of orientation (6, 9, 45, 60, and 90° tilted from vertical) and luminance (10.2, 11.8, 17.9, 21.5, and 30.0 cd/m^2^) contrasts to estimate search efficiency of the stimuli in this paradigm. Whereas set sizes (9 and 21 possible target locations) and contrasts were mixed within blocks, orientation and luminance dimensions were blocked and block sequence was counterbalanced across participants. Search displays, stimuli, target positions as well as the procedure and task were the same as in the *Baseline salience measurement*, except that the midpoint of the small set size display was randomly placed on 8 positions of an imaginary circle of 1.88° radius. Participants completed 20 blocks à 80 trials, providing 80 trials per contrast condition.

Eight different participants (4 male, median age 25 [range: 19 - 49] years, all dextral and with corrected-to-normal vision) took part in the pilot experiment for € 8 or course credit. Trials with RTs faster than 150 and slower than 1,000 ms were excluded from analysis (1.5 %), as were practice trials (the first 10 of the first two blocks and first 3 of the following blocks). Trials with response errors (2.3%) were also excluded from analysis.

For the remaining trials, slopes and intercepts of the functions relating RT to set size were calculated for each contrast condition. Here, slopes of the search function indicate the time that is required to shift attention from one item to another and intercepts indicate the time for non-search processes. The criteria of Wolfe and Horowitz [2] were applied, according to which slopes near zero ms/item indicate efficient search and slopes of 20 ms/item and greater indicate inefficient search. Table S1 summarizes the slope and intercept results of the pilot experiment.

Based on the results, we chose contrasts for the distractor experiment. Because slopes were all quiet shallow, indicating efficient searches, we based our choice on intercepts, avoiding the slowest ones.

# **References**

1. Cave KR, Wolfe JM (1990) Modeling the role of parallel processing in visual search. Cogn Psychol 22: 225–271.
2. Wolfe JM, Horowitz TS (2004) What attributes guide the deployment of visual attention and how do they do it? Nat Rev Neurosci 5: 1–7.
