# Supplementary material for: Salience-Based Selection: Attentional Capture by Distractors Less Salient Than the Target
Source: PLoS One. 2013 Jan 28;8(1):e52595. doi: 10.1371/journal.pone.0052595 (PMC3557287; doi:10.1371/journal.pone.0052595)
Supplement: Table S1 — Slopes and intercepts of the search RT functions for orientation and luminance contrast conditions. (DOCX) [file pone.0052595.s002.docx]

# **Table S1**

# **Slopes and intercepts of the search RT functions for orientation and luminance contrast conditions**

| **Contrast** | **Slope (ms/item)** | **Intercept (ms)** |
| --- | --- | --- |
| *Orientation (°)* |  |  |
| 6 | 1.8 | 531 |
| 9 | 1.3 | 434 |
| 45 | 0.2 | 377 |
| 60 | 0.7 | 370 |
| 90 | 1.0 | 377 |
| *Luminance (cd/m^2^)* |  |  |
| 10.2 | 3.8 | 540 |
| 11.8 | 4.1 | 480 |
| 17.9 | 2.1 | 420 |
| 21.5 | 1.8 | 396 |
| 30.0 | 1.9 | 372 |
